# Exploring novel markers for coronary heart disease associated with systemic lupus erythematosus: A review

**DOI:** 10.1097/MD.0000000000040773

**Published:** 2024-12-13

**Authors:** Linping Du, Yuqun Wang, Honglei Ma, Jiaheng Fan, Shiqi Wang, Junhong Liu, Xiaodong Wang

**Affiliations:** a Affiliated Hospital of Shandong Second Medical University, School of Clinical Medicine, Weifang, China; b Shandong Second Medical University, Weifang, China.

**Keywords:** biomarkers, coronary heart disease, systemic lupus erythematosus

## Abstract

Systemic lupus erythematosus (SLE) is an autoimmune condition that is characterized by the production of autoantibodies and sustained inflammatory damage. Coronary heart disease (CHD) is a common complication of SLE, significantly increases CHD-related mortality in SLE patients. Despite conventional risk factors, the mechanisms contributing to a higher CHD risk require further investigation, with the immune and inflammatory aspects of SLE playing a significant role. Endothelial cell damage and dysfunction are key factors in the progression of coronary atherosclerosis in SLE patients. This review specifically focuses on endothelial dysfunction and the role of specific microRNAs in the context of SLE and CHD. In addition, we discuss the effects and functions of oxidative stress markers, endothelial progenitor cells, and circulating endothelial cells in individuals with both SLE and CHD. We also explored the typical inflammatory markers associated with SLE and CHD, addressing their clinical significance and limitations.

## 1. Introduction

Systemic lupus erythematosus (SLE) encompasses a spectrum of complex autoimmune diseases that affect multiple organs and systems in the body.^[1]^ Characterized by a dysregulated immune response, SLE leads to the body’s defense mechanisms indiscriminately targeting its tissues and cells.^[1]^ This chronic condition presents with a broad array of symptoms and complications that pose significant medical challenges. The relationship between SLE and coronary heart disease (CHD), the leading cause of morbidity and mortality worldwide, is particularly notable. SLE patients are not only at an increased risk of developing CHD compared to the general population, but also face a higher mortality rate due to more aggressive disease progression, including accelerated atherosclerosis, which is a condition where plaque builds up inside the arteries at an enhanced rate due to chronic inflammation and immune system dysregulation specific to SLE.^[2]^

The pathogenesis of SLE involves a variety of factors including genetic predispositions, environmental triggers such as ultraviolet light, and immune system abnormalities.^[3]^ Moreover, aberrant immune responses influenced by epigenetic factors such as microRNA (miRNA) contribute to this complexity.^[4]^ Similarly, the development of CHD in patients with SLE is influenced not only by traditional risk factors, such as age, sex, hypertension, hyperlipidemia, smoking, diabetes, obesity, and family history, but also by SLE-specific immune-related factors that exacerbate vascular damage through mechanisms such as endothelial dysfunction and enhanced inflammatory responses (Table 1).

**Table 1:** Traditional and SLE-specific risk factors.

Risk factors for traditional CHD	SLE-specific risk factors
Age	Disease activity and duration in SLE
Sex	Abnormal adaptive immune response
Hypertensive	Pro-inflammatory cytokine signaling
Hyperlipidemia	Elevated type 1 interferon
Smoking	Dysregulation of neutrophil extracellular traps
Diabetes	Oxidative stress
Obesity	Autoantibody
Family history of CHD	High-density lipoprotein dysfunction

CHD = coronary heart disease, SLE = systemic lupus erythematosus.

**Figure 1.**
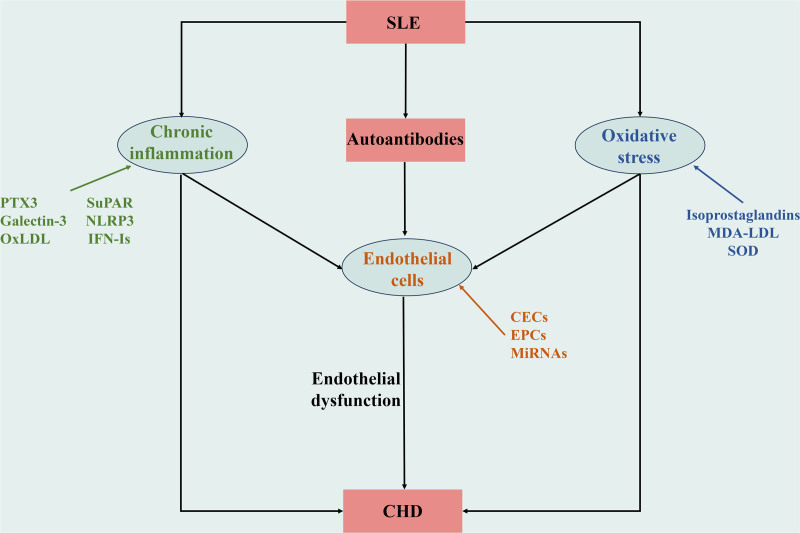
Endothelial cells, chronic inflammation, and oxidative stress promote SLE complicated with CHD. Autoantibodies, chronic inflammation, and oxidative stress in SLE patients act on endothelial cells, damaging endothelial cells, and promoting endothelial dysfunction. The changes of biomarkers related to endothelial cells, chronic inflammation, and oxidative stress reflect their participation, which together promote SLE complicated with CHD. CHD = coronary heart disease, SLE = systemic lupus erythematosus.

Endothelial cells, which form a critical interface between the circulating blood and vascular tissues, play a pivotal role in maintaining physiological functions and vascular integrity. In patients with SLE, these cells are often the site of significant damage due to persistent inflammation and oxidative stress, leading to compromised vascular health and accelerated atherogenesis. Chronic inflammation and oxidative stress are not only prevalent in SLE, but are also crucial contributors to the onset and progression of CHD in these patients (Fig. 1).

This review delves into the intricate relationship between SLE and CHD, emphasizing the unique pathophysiological pathways and the role of accelerated atherosclerosis in patients with SLE. By exploring this multifaceted connection, this review highlights potential clinical treatment strategies that could mitigate CHD risk and improve the overall prognosis of patients with SLE.

## 2. Endothelial dysfunction and CHD in SLE

### 2.1. Role of endothelial cells in vascular function

Endothelial cells are central to the relationship between SLE and CHD. These cells comprise the inner lining of the blood vessels and serve as gatekeepers that regulate vascular function. Endothelial cells serve as critical regulatory hubs within the cardiovascular homeostatic network and play a crucial role in maintaining vascular health and function. In addition to their structural role, endothelial cells maintain vascular homeostasis by controlling blood flow, preventing thrombosis, and promoting an anti-inflammatory environment.^[5]^ Moreover, they actively participate in maintaining the delicate balance between pro-thrombotic and antithrombotic factors, while also contributing to the production of nitric oxide (NO). NO is essential for preserving normal blood flow and preventing arterial plaques.^[6]^ Endothelial cells are responsible for regulating processes, such as vasodilation and vasoconstriction, blood coagulation control, and modulation of inflammation.

### 2.2. Impact of endothelial dysfunction on the development of CHD in SLE patients

SLE-related endothelial dysfunction may be a predisposing factor for CHD in these patients.^[7]^ This represents an early event in atherosclerosis, characterized by the reversible loss of vessel homeostasis. Endothelial dysfunction is a unique hazard indicator of SLE and accelerates disease progression.^[8]^ Impaired endothelial function contributes to the establishment of a pro-inflammatory and pro-thrombotic environment within the vasculature. This inflammatory milieu expedites the formation of atherosclerotic plaques and increases the risk of plaque rupture, which subsequently leads to thrombosis. Moreover, vascular endothelial dysfunction affects vasodilation, ultimately contributing to hypertension, a primary cardiac risk factor.^[9,10]^ Consequently, patients with SLE with endothelial dysfunction are trapped in a vicious cycle. Endothelial disorders increase susceptibility to heart disease, underscoring the need for a comprehensive understanding to eventually develop targeted interventions.

### 2.3. Chronic inflammation and endothelial dysfunction in SLE combined with CHD

Patients with SLE exhibit a dysregulated immune system that results in chronic inflammation. Persistent inflammation triggers endothelial dysfunction. Inflammatory mediators released during SLE activate endothelial cells, leading to the release of adhesion molecule, which in turn accelerate the adherence of immune cells to the endothelium. This heightened immune cell adhesion disrupts endothelial cell function, impairing their ability to regulate vascular tone and inflammation.^[11]^ However, in the context of SLE, the release of inflammatory mediators, vascular endothelial cytokines, and autoantibodies leads to the dysfunction of vascular endothelial cells.^[12]^ Endothelial dysfunction has been shown to contributes to the development of atherosclerosis.^[13]^ In SLE, the inflammatory response activates immune cells to produce reactive oxygen species (ROS), which in turn damage tissues and disrupt immune regulation. ROS also stimulate the release of inflammatory cytokines from endothelial cells through the oxidation of low-density lipoprotein (oxLDL). These cytokines promote chronic inflammation, leading to endothelial dysfunction and atherosclerosis. Persistent inflammation promotes immune cell infiltration, oxidative stress, and damages endothelial integrity and vasomotor function. Furthermore, inflammation is a key factor that drives the initiation, formation, and eventual rupture of atherosclerotic plaques.^[14]^ Suppression of inflammation is a treatment strategy to reduce CHD risk in patients with SLE.^[12]^

### 2.4. Oxidative stress and endothelial dysfunction in SLE combined with CHD

Oxidative stress is a central factor that promotes the progression of both SLE and CHD, leading to endothelial dysfunction and contributing to atherosclerosis development of atherosclerosis.^[15]^ Oxidative stress is a common pathology in T cell-mediated autoimmune diseases. Oxidative stress is an imbalance between the production and clearance of ROS, and underlies abnormal exposure and response to cell death signals.^[16]^ Abnormal cell death signaling triggers the immune system, causing apoptotic and necrotic cells to release nuclear debris and stimulate the production of antinuclear antibodies. Antinuclear antibodies bind to antigens in the endothelial cells to form immune complexes. Immune complexes are deposited in the vessel wall, triggering a chronic inflammatory response and endothelial disorders.^[17]^ Excessive oxidative stress increases ROS levels, intracellular calcium concentration, ATP consumption, mitochondrial load, and apoptosis.^[18]^ Excessive oxidative stress activates the nuclear factor κ-B (NF-κB) signaling pathway in endothelial cells, which produces a variety of pro-inflammatory factors, such as tumor necrosis factor-α (TNF-α) and matrix metalloproteinases (MMPs), contributing to endothelial dysfunction and lipid plaque formation.^[19]^

## 3. Endothelial cell biomarker biomarkers and their significance

### 3.1. Endothelial progenitor cells (EPCs) as biomarkers

EPCs are subpopulations of circulating stem cells that contribute to endothelial repair and angiogenesis. EPCs are implicated in the regeneration of damaged vascular endothelium and formation of neointima, which is essential for maintaining vascular health. In cases of vascular damage, EPCs are mobilized from the bone marrow, enter the bloodstream, and are transported to the injury site, where they aid in endothelial recovery. In SLE-associated CHD, EPC function is impaired owing to the complex interplay of various factors. Chronic inflammation, oxidative stress, and autoimmune dysregulation contribute to EPC dysfunction in SLE. The inflammatory environment in SLE leads to reduced EPC proliferation, impaired migration to the site of injury, and compromised endothelial repair. Additionally, oxidative stress adversely affects the survival and function of EPCs, thereby hindering their ability to regenerate.^[20]^ Monitoring EPC levels and function serves as an indicator for evaluating disease progression and treatment response in SLE-associated CHD.^[21]^ Decreased EPC numbers and impaired function indicate ongoing endothelial damage and dysfunction. EPCs play a direct role in endothelial repair mechanisms, and their diminished capacity reflects the compromised regenerative potential of the endothelium in SLE patients.^[22]^ Moreover, efforts targeting the restoration of EPC function have the potential to improve endothelial dysfunction and lower CHD risk.

### 3.2. Circulating endothelial cells (CECs) as biomarkers

CECs are shed into the bloodstream during episodes of endothelial injury and dysfunction. Disruption of the endothelial barrier results in the release of CECs from the vascular endothelium, enabling their entry into circulation.^[23]^ In healthy individuals, the presence of CECs is minimal. However, CEC levels were notably elevated in conditions marked by significant endothelial damage, such as SLE-associated CHD. Increased CEC levels directly indicate ongoing endothelial damage and dysfunction. The heightened release of CECs in these patients serves as a signal of the extent of endothelial damage and reflects the severity of vascular involvement as well as the likelihood of cardiovascular complications.^[24]^ Monitoring changes in the levels of CECs by detecting cells positive for CD146 cells that stained for annexin V serves as a potential noninvasive marker for assessing endothelial health and disease activity in SLE patients.^[25,26]^ Apoptosis of CECs leads to the production of phospholipid-rich particles that elevate tissue factor levels thereby increasing the central involvement of endothelial injury in the pathogenesis of vascular abnormalities in SLE.^[27]^ Detection of tissue factor levels can reflect apoptosis in CECs to track disease progression.

### 3.3. MiRNAs as emerging markers

MiRNAs are endogenous noncoding RNAs that modulate the expression of relevant gene. They accomplish this by binding to specific messenger RNAs, resulting in either messenger RNA degradation or translation inhibition. miRNAs have been implicated in diverse physiological processes, including cell polarization, reproduction, and immune responses.^[28]^ Recent studies have highlighted specific miRNAs with altered expression in damaged endothelial cells. For instance, miR-92 is upregulated in patients with SLE and is related to increased endothelial cell apoptosis and dysfunction.^[29]^ Conversely, miR-126 promotes endothelial cell repair and angiogenesis, but is downregulated in SLE patients with endothelial cell dysfunction and impaired vascular repair mechanisms.^[30]^

Dysregulation of miRNAs in SLE-associated endothelial dysfunction holds promise for the development of diagnostic and prognostic markers. miRNAs govern pathophysiological processes, including cell adhesion, proliferation, lipid uptake and efflux, and generation of inflammatory mediators. miRNAs can be detected in multiple body fluids, making them attractive candidates for noninvasive diagnostic testing.^[31]^ Furthermore, the unique miRNA expression profile associated with endothelial dysfunction in SLE serves as a prognostic indicator that facilitates the identification of patients with CHD.

## 4. Oxidative stress biomarkers and their significance

### 4.1.1. Isoprostaglandins

Isoprostaglandins are prostaglandin-like compounds formed by the peroxidation of polyunsaturated fatty acids. They also promote vasoconstriction, smooth muscle cell proliferation, and platelet aggregation. Isoprostaglandins are stable molecules that are present in all tissues, plasma, and urine. Elevated levels of isoprostaglandins in SLE patients indicate increased oxidative stress.^[32]^ They serve as reliable markers of lipid peroxidation and reflect the extent of the oxidative damage. Studies have reported increased levels of F2 isoprostaglandins (F2IsoP, a highly stable isoprostaglandin) in macrophages and smooth muscle cells within atherosclerotic plaques of patients with CHD. This increase in F2IsoP levels suggests a strong association between oxidative stress and atherosclerosis. LeLeiko et al^[33]^ found elevated serum F2IsoP levels in 108 patients with CHD. Isoprostaglandin levels are not affected by the lipid content of the diet. Quantitative analysis of isoprostaglandins in the plasma or urine may reflect oxidatively mediated vascular involvement in SLE patients.^[34]^ Isoprostaglandin measurement contributes to the understanding of the causal role of oxidative damage in vascular diseases of SLE.^[35]^ Mass spectrometry and enzyme-linked immunosorbent assay can be used to accurately measure isoprostaglandins in biological samples. In vivo formation increases as a function of lipid peroxidation and isoprostane is very stable and can be easily measured independent of dietary lipid content. Isoprostaglandins are present in all healthy tissues and biological fluids in detectable amounts that allow for the definition of reference intervals and are therefore well suited as biomarkers.^[36]^

### 4.1.2. Malondialdehyde-modified low-density lipoprotein (MDA-LDL)

Increased levels of MDA-LDL are linked to confluence of SLE and CHD. Oxidative stress in patients with SLE enhances the formation of MDA-LDL, promoting endothelial dysfunction and atherosclerosis. MDA is a stable aldehyde produced in vivo through lipid peroxidation and serves as a widely available oxidative stress biomarker. MDA-LDL damages the blood vessels, increases the expression of growth factors, promotes lipid accumulation, and contributes to plaque instability in the vessel wall.^[37]^ Immune complexes involving MDA-LDL promote macrophage apoptosis and inhibit cell proliferation. Additionally, the MDA-LDL complex induces the release of matrix MMPs and TNF-α. TNF-α release increases the expression of vascular cell adhesion molecules 1, intercellular cell adhesion molecule-1, and E-selectin thereby promoting inflammation, increasing monocyte and vascular endothelial cell interactions between monocytes and vascular endothelial cells, and inducing endothelial cell apoptosis.^[38]^ MMPs thin the fibrous cap of atherosclerotic plaques. A combination of apoptosis and fibrous cap thinning increases the risk of atherosclerotic plaque rupture.^[39]^ Measurement of MDA-LDL levels provide insights into the extent of lipid peroxidation and oxidative damage.

#### 4.1.3. Superoxide dismutase (SOD) isoforms

SOD isoforms are antioxidants that neutralize superoxide radicals. There are 3 isoforms of SOD, with SOD3 (extracellular SOD) being predominant in the vascular tissue. In SLE patients, SOD isoforms undergo constant changes, especially in the ratio of MnSOD to CuZnSODs.^[40]^ Dysregulation of SOD isoenzymes disrupts the balance between ROS production and scavenging, intensifying oxidative stress, and endothelial dysfunction. Moreover, overexpression of SOD3 in animal models attenuates tissue damage caused by ischemia.^[41]^ Vascular wall intrinsic progenitor cells produce and secrete substantial amounts of SOD3 to mitigate oxidative stress-induced endothelial cell injury.

### 4.1. Indicators of vascular damage and cardiovascular risk

**Figure 2.**
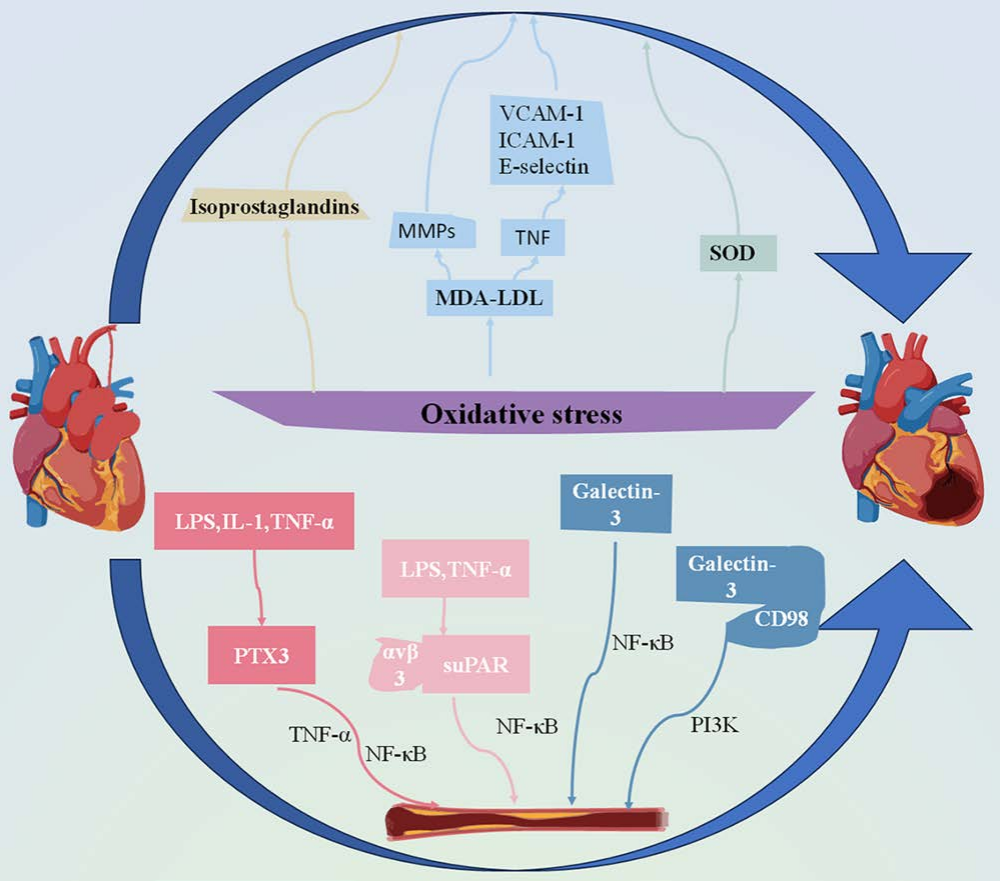
Mechanism of oxidative stress and inflammatory response leading to CHD associated with SLE. In the state of oxidative stress, the dysregulation of peroxide isoprostaglandins, MDA-LDL and antioxidant SOD promote the occurrence of atherosclerosis. In the inflammatory environment, macrophages produce PTX3 when stimulated by LPS, IL-1, and TNF-α. PTX3 co-promotes the occurrence of atherosclerosis with TNF-α/NF-κB. Under the stimulation of LPS and TNF-α, SuPAR can bind to αvβ3 integrins and promote atherosclerosis through NF-κB pathway. When the body is in inflammatory response, the expression of Galectin-3 is increased, which can accelerate the process of atherosclerosis through the NF-κB pathway or the PI3K pathway after binding with CD98. IL-1 = interleukin-1, LPS = lipopolysaccharide, MDA-LDL = malondialdehyde-modified low-density lipoprotein, NF-Κb = nuclear factor kappa-B, PI3K = phosphatidylinositol 3-kinase, PTX3 = pentatoxin-3, SOD = superoxide dismutase, SuPAR = soluble urokinase-type plasminogen activator receptor, TNF-α = tumor necrosis factor-α.

These oxidative stress biomarkers serve as indicators of vascular injury and cardiovascular risk in SLE patients. Elevated levels of isoprostaglandins signify ongoing lipid peroxidation, a process closely linked to endothelial dysfunction and atherosclerosis progression. Oxidative modification of LDL is associated with plaque formation and vascular inflammation, exacerbating CHD in patients with SLE.^[42,43]^ Dysregulation of SOD isoforms results in impaired antioxidant defence mechanisms, further amplifying oxidative stress and contributing to endothelial dysfunction^[44]^ (Fig. 2).

## 5. Novel inflammatory biomarkers of SLE-associated CHD

### 5.1.1. Pentatoxin-3 (PTX3)

PTX3 is a multimeric acute phase protein. PTX3 is produced in macrophages stimulated by lipopolysaccharide (LPS), interleukin-1 (IL-1), and TNF-α.^[45]^ PTX3 plays a role in mediating innate immune responses and inflammatory processes. Elevated PTX3 levels have been observed in patients with SLE, especially those with cardiovascular complications. PTX3 exacerbates vascular damage in SLE through the synergistic acceleration of TNF-α/NF-κB signaling and DNA damage response. TNF-α activates NF-κB in vivo, which upregulates PTX3 expression.^[46]^ After TNF-α treatment, NF-κB is activated and translocates from the cytoplasm to the nucleus, where it binds to the corresponding DNA fragment to regulate the transcription and expression of PTX3. The upregulation of PTX3 reflects an increased inflammatory burden and is a potential predictor of endothelial dysfunction and the development of CHD in SLE patients.^[47]^

### 5.1.2. Soluble urokinase-type plasminogen activator receptor (SuPAR)

SuPAR is a chronic inflammatory biomarker linked to activation of the innate immune system. Elevated suPAR levels have been linked to immune dysregulation and endothelial dysfunction in SLE patients.^[48]^ LPS and TNF-α induce suPAR release by stimulating human peripheral blood mononuclear cells and neutrophils. SuPAR binds to integrins, particularly the αvβ3 heterodimer adhesion receptor, initiating endothelial cell atherosclerosis, and promoting inflammation via the NF-κB pathway. A study by Ghasemzadeh et al^[49]^ measured suPAR levels in 3278 CHD patients and found that suPAR levels ≥ 3.5 ng/mL were associated with mortality in CHD patients.

### 5.1.3. Galectin-3

Increased galectin-3 levels are associated with endothelial dysfunction and atherosclerosis in patients with SLE. Galectin-3 is a carbohydrate-binding protein and endogenous immunomodulator secreted by immune cells. Galectin-3 is abnormally expressed during inflammatory reactions in vivo and regulates the severity of inflammation.^[50]^ Galectin-3 induces the activation of the NLRP3-dependent pathway under different acute and/or chronic pathological conditions. It also promotes type I interferons (IFN-Is) responses and increases serum IFN-γ levels, thereby accelerating the onset and progression of SLE.^[51]^ Galectin-3 binds to CD98, a type II transmembrane heterodimer, and enhances IL-4-mediated macrophage activation through the PI3K pathway. This promotes monocyte migration and macrophage infiltration of the arterial wall, exacerbating the pro-inflammatory state of atherosclerosis.^[52]^

### 5.1.4. NLR family Pyrin domain-containing protein 3 (NLRP3)

**Figure 3.**
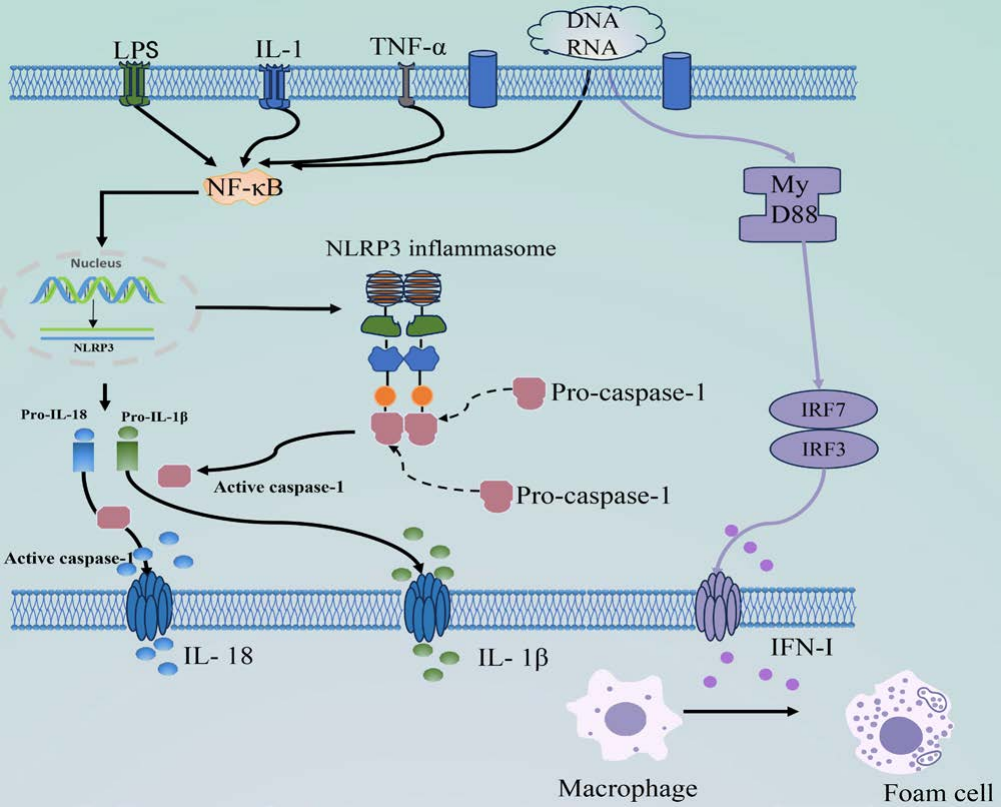
The initiation and activation of inflammasome of NLRP3, and the production process of IFN-I. Specific PRRS (such as LPS, IL-1, and TNF-α) activate the NF-κB signaling pathway by recognizing PAMPs, and the body’s own DNA or RNA by recognizing DAMPs. Activation of NF-κB signaling causes NLRP3, pro-IL-1β, and pro-IL-18 to upregulate and activate NLRP3 inflammasome assembly, recruiting and activating pro-caspase-1. Activated caspase-1 cleaves the pro-inflammatory cytokines pro-IL-1β and pro-IL-18 into mature IL-1β and IL-18. RNA or DNA can also turn on the downstream MyD88, which increases the expression of IRF-3 and IRF-7 leading to IFN-I production, and IFN-1 prompts macrophages to become foam cells. DAMPs = damage-associated molecular patterns, IFN-I = type I interferon, IL-1β = interleukin-1β, IL-18 = interleukin-18, IL-1 = interleukin-1, IRF-3 = interferon regulatory factors-3, IRF-7 = interferon regulatory factors-7, LPS = lipopolysaccharide, MyD88 = myeloid differentiation factor 88, NF-Κb = nuclear factor kappa-B, NLRP3 = NLR family Pyrin domain-containing protein 3, PAMPs = pathogen-associated molecular patterns, PRRS = pattern recognition receptors, Pro-IL-1β = pro-interleukin-1β, Pro-IL-18 = pro-interleukin-18, TNF-α = tumor necrosis factor-α.

The presence of NLRP3 indicates inflammation and initiation of atherosclerosis in patients with SLE. Innate immune cells express nucleotide-binding leucine-rich repeat receptors (NOD-like receptors, NLR) with a structural pattern recognition receptor structural domain.^[53]^ These receptors generate inflammatory responses by recognizing 2 molecular patterns. Activated NLRP3 inflammasomes contribute to endothelial damage and induce atherosclerosis development of atherosclerosis.^[54]^ The full activation of NLRP3 inflammasomes involves 2 steps: an initial triggering phase and subsequent assembly and activation of the inflammasome complex. Activated NLRP3 inflammasomes drive atherogenesis and progression of atherosclerosis through the NF-κB signaling pathway, producing Caspase-1, IL-1β, and IL-18.^[55]^ Activation of NLRP3 inflammatory corpuscles also leads to a systemic inflammatory response, indirectly stimulating neutrophil activation. Activated neutrophils enter atherosclerotic plaques and disrupt the balance of neutrophil extracellular traps (NET)^[56]^ (Fig. 3).

### 5.1.5. Oxidized low-density lipoprotein (OxLDL)

OxLDL is the oxidized form of low-density lipoprotein (LDL). LDL subjected to oxidative stress and inflammatory stimuli undergoes spontaneous or enzymatic oxidation to form oxLDL. OxLDL activates endothelial cells and monocytes. Monocytes maintain local inflammatory responses by secreting pro-inflammatory cytokines, chemokines, and reactive oxides.^[57]^ The development of atherosclerosis is strongly associated with the inflammatory processes in macrophages. In the early stages, excessively oxLDL infiltrates endothelial cells and causes oxidative damage to the endothelium. OxLDL then induces the expression of endothelial cell adhesion molecules and recruits monocytes that subsequently differentiate into macrophages.^[58]^ OxLDL combines with human macrophages, such as THP-1 cells, leading to the secretion of growth factors and extracellular matrix components. These growth factors and extracellular matrix proteins promote the formation of vascular atheromatous plaques.^[59]^ OxLDL also activates toll-like receptors, triggering pro-inflammatory signaling pathways that induce the secretion of IL-1β, IL-6, or TNF-α, leading to a chronic inflammatory response. OxLDL also activates macrophages, resulting in foam cells formation. Foam cells are a major component of atherosclerotic plaques, and their accumulation contributes to lipid storage within the plaque and continued plaque growth.^[60]^

### 5.1.6. IFN-Is

IFN-Is are triggered by pathogen-related molecular patterns. Plasmacytoid dendritic cells detect pathogenic RNA or DNA via receptor-mediated endocytosis, initiating downstream signaling molecules, such as myeloid differentiation factor 88 and subsequent transcription factors. The expression of downstream transcription factors such as interferon regulatory factor (IRF)-3 and IRF-7 results in IFN-I production.^[61]^ IFN-I inhibits NO secretion from vascular endothelial cells in patients with SLE and promotes vascular endothelial dysfunction. Endothelial dysfunction facilitates the development of atherosclerosis in SLE patients. IFN-I also hinders vascular repair and inhibits angiogenesis by reducing endothelial progenitor levels and inhibiting neointimal formation at sites of endothelial injury.^[62]^ IFN-I contributes to the atherosclerotic process by enhancing foam cell formation and regulating macrophages and cytotoxic T cells. Furthermore, it promotes platelet activation and increases the risk of plaque rupture^[63]^ (Fig. 3).

### 5.1. Potential as indicators of inflammatory burden and vascular risk

These novel inflammatory biomarkers have the potential to serve as indicators of the inflammatory burden and vascular risk in SLE-associated CHD. Elevated levels of PTX3, suPAR, galectin-3, NLRP3, macrophages, IFN-I, and NETs directly reflected the degree of immune activation and inflammation. The presence of these novel inflammatory biomarkers indicates an environment conducive to endothelial dysfunction, atherosclerosis, and an increased cardiovascular risk. By incorporating these markers into clinical evaluations, we can gain a more comprehensive understanding of the role of the inflammatory process. These markers provide valuable insights into the likelihood of cardiovascular complications in patients with SLE.

## 6. Clinical implications and future directions

### 6.1. Potential clinical implications of novel markers for SLE-associated CHD

Incorporating these novel markers into clinical practice holds significant clinical implications for SLE-associated CHD. Healthcare providers can tailor treatment strategies for their patients by utilizing a range of markers, including oxidative stress biomarkers, EPCs, CECs, and inflammatory markers. Measurement of these markers can more accurately assess disease severity and progression.

### 6.2. Prospects for diagnostic and therapeutic guidance

The application of these markers shows promise in enhancing risk stratification, early diagnosis, and treatment guidance for SLE patients at risk of developing heart disease. Comprehensive insights gained from these markers will aid in identifying individuals with a higher likelihood of endothelial dysfunction and cardiovascular events. Developing early intervention strategies based on these markers that target the underlying mechanisms may mitigate disease progression and improve prognosis.

### 6.3. Limitations

There is a need for longitudinal studies to explore the relationship between CHD and EPC damage in patients with SLE. Clinical evidence supporting the role of EPCs in the pathogenesis of coronary artery disease in SLE patients is currently limited, and further research is required to investigate many of the potential markers of SLE-associated coronary artery disease. Although these markers may provide distinct therapeutic options for disease management, the development of drugs targeting these markers is still pending.

## 7. Conclusion

The intricate interplay between SLE and CHD underscores the critical role of endothelial dysfunction in connecting these 2 health challenges. Endothelial dysfunction, characterized by impaired endothelial cell function and integrity, serves as a common denominator contributing to the heightened risk of CHD in SLE patients. Chronic inflammation, oxidative stress, and immune dysregulation inherent to SLE collectively lead to impaired endothelial function, setting the stage for the development of atherosclerosis and subsequent cardiovascular complications. Oxidative stress biomarkers EPCs, CECs, and inflammatory biomarkers provide a comprehensive molecular perspective of the disease process. These markers offer valuable insights into the mechanisms underpinning endothelial dysfunction and cardiovascular risk. Their incorporation enables precise risk assessment and the formulation of rational management strategies. By integrating these markers into clinical practice, healthcare providers can identify populations at risk, make informed treatment decisions, and decelerate the progression of endothelial dysfunction.

## Author contributions

**Conceptualization:** Linping Du, Xiaodong Wang.

**Data curation:** Junhong Liu.

**Funding acquisition:** Honglei Ma.

**Investigation:** Junhong Liu.

**Project administration:** Honglei Ma.

**Resources:** Yuqun Wang.

**Software:** Jiaheng Fan.

**Supervision:** Jiaheng Fan.

**Validation:** Yuqun Wang.

**Visualization:** Shiqi Wang.

**Writing – original draft:** Linping Du, Xiaodong Wang.

**Writing – review & editing:** Xiaodong Wang.
